# Generalization of Optimal Control Saturation Pulse Design for Robust and High CEST Contrast

**DOI:** 10.1002/mrm.70150

**Published:** 2025-10-28

**Authors:** Clemens Stilianu, Markus Huemer, Moritz Zaiss, Rudolf Stollberger

**Affiliations:** ^1^ Institute of Biomedical Imaging Graz University of Technology Graz Austria; ^2^ Institute of Neuroradiology Friedrich‐Alexander‐Universität Erlangen‐Nürnberg (FAU), University Hospital Erlangen Erlangen Germany; ^3^ High‐Field Magnetic Resonance Center Max‐Planck Institute for Biological Cybernetics Tübingen Germany; ^4^ BioTechMed Graz Graz Austria

**Keywords:** contrast mechanism, open source, optimal control, pulse design, pulsed CEST

## Abstract

**Purpose:**

Optimal control (OC) chemical exchange saturation transfer (CEST) pulse design for singular pulses that can be used flexibly and robustly with high saturation at different duty cycles, saturation durations, and magnetic field strengths.

**Theory and Methods:**

An OC framework was developed to design a single‐pulse shape that can be flexibly applied for arbitrary pulse train parameters and outperform typically used CEST saturation pulse shapes. The pulse design was developed primarily with a continuous‐wave (CW) spectrum as the optimization target, but can be easily adapted to specific scenarios. The generalized OC pulse was evaluated through simulations, a phantom, and in vivo measurements on a 3 T clinical scanner. Performance was assessed in terms of contrast, robustness to field inhomogeneities, and resilience against artifacts such as Rabi oscillations and sidebands, compared to established saturation techniques.

**Results:**

Investigations showed that the generalized OC pulse achieved a contrast matching CW saturation and also functioned well under field inhomogeneities. Low‐pass filtering of the optimized pulse shape effectively suppressed artifacts outside the initial optimization frequency range, enabling generalization across different field strengths. Phantom experiments consistently showed higher contrast than Gaussian, Fermi, and adiabatic spin‐lock (aSL) pulses for various CEST agents covering most clinically relevant regimes. In vivo imaging demonstrated substantially enhanced CEST contrast for both creatine/phosphocreatine in muscle and amide proton transfer (APT) in the brain compared to Gaussian saturation.

**Conclusion:**

The generalized OC pulse provides a robust and flexible alternative to conventional CEST saturation strategies. Its integration into the open‐source Pulseq‐CEST framework supports simple reproducibility and a vendor‐independent implementation.

## 1 Introduction

A conventional chemical exchange saturation transfer (CEST) experiment uses off‐resonant RF saturation pulses, followed by an imaging sequence repeated at multiple frequencies to generate a Z‐spectrum. While continuous‐wave (CW) saturation is common in preclinical settings due to its high exchange weighting and robust spectra, it is not feasible or reliable on clinical systems due to hardware limitations. Pulsed saturation is typically used in clinical systems, but is prone to Rabi oscillations [1] and sideband artifacts [2], which are influenced by pulse shape. Additionally, the CEST contrast is highly dependent on the chosen pulse shape and usually lower than with CW saturation [3, 4, 5, 6].

Pulsed‐CEST saturation can also be used to enhance the temporal resolution of CEST experiments by interleaving pulses with image acquisition. This approach allows further use of magnetization dynamics for studying exchange rates, $T_{1}$ mapping, or motion correction [7, 8, 9, 10, 11, 12]. Furthermore, pulsed CEST is required for parallel transmit $B_{1}$ shimming [13, 14, 15].

RF pulse shape design has been investigated by various groups in the past. Rancan et al. [16] applied gradient‐based optimization for free‐form RF pulse design targeting paramagnetic CEST contrast agents with high exchange rates in preclinical settings. Yoshimaru et al. [17] employed metaheuristic multi‐objective optimization to design pulsed‐CEST saturation based on a Fourier series for clinical applications. More recently, Mohanta et al. (2024) used gradient‐based optimization to design 100 ms pulses for high exchange rate and high chemical shift CEST contrast agents at 11.7 T in preclinical settings.

Off‐resonant spin‐lock (SL) experiments extend conventional pulsed‐CEST saturation. Jin and Kim [18]. The optimization of adiabatic pulses for SL measurements has improved robustness against field inhomogeneities, enabling high exchange weighting and stable SL saturation for imaging [19, 20, 21, 22, 23, 24, 25, 26]. However, adiabatic tipping pulses are time‐ and energy‐intensive. Adiabatic pulses still have limited robustness to field inhomogeneities, with performance rapidly degrading outside defined $B_{0}$ and $B_{1}$ tolerance ranges [6, 19, 27, 28].

A recent study shows that CEST saturation pulse trains optimized by optimal control (OC) offer superior contrast and robustness over Gaussian, block pulse train (BPT), and adiabatic spin‐lock (aSL) techniques for equivalent saturation times (Tsat) and Duty Cycle (DC) [6]. However, these Fully Optimized Pulse Trains (FOPTs) have a fixed $T_{\text{sat}}$, and while combining pulse trains can extend $T_{\text{sat}}$, they lack the flexibility of single pulses (e.g., Gaussian pulses) in being applied with arbitrary DC and $T_{\text{sat}}$.

This work investigates a generalization of the OC‐based saturation pulse design to allow high flexibility for CEST experiments. The goal is to develop a versatile pulse that operates effectively across various duty cycles, pulse times, total saturation times, and $B_{0}$ field strengths. Furthermore, we investigate whether pulses that have been designed for a specific duration can be used with different pulse durations by scaling them accordingly.

We evaluate the generalized pulse under varying conditions in both simulations and phantom experiments. Additionally, the OC performance is tested in a thigh muscle creatine (Cr) and phosphocreatine (PCr) contrast, as well as in a brain amide proton transfer (APT) model. The resulting pulses are integrated into the open‐source Pulseq‐CEST framework [29, 30], enabling reproducibility and easy implementation across different scanners.

## 2 Methods

### 2.1 RF Pulse Design

The CEST saturation pulses are designed by minimization of the objective function J:

(1) $$\begin{array}{ll} \underset{B_{1}(t)}{\min}\; & J(B_{1}(t),M_{z}(\omega ,t),{\tilde{M}}_{z}(\omega ,t))=\frac{\alpha }{2}\overset{T_{\text{sat}}}{\underset{t=0}{\int }}B_{1}{\left(t\right)}^{2}dt\\ & \;+\frac{\sigma }{p}\underset{\omega }{\sum }{\left|\frac{M_{z}(\omega ,T_{\text{sat}})-M_{zdes}(\omega )}{\epsilon }\right|}^{p}\\ & \;+\frac{\sigma }{p}\underset{\omega }{\sum }{\left|\frac{{\tilde{M}}_{z}(\omega ,T_{\text{sat}})-{\tilde{M}}_{zdes}(\omega )}{\epsilon }\right|}^{p} \end{array}$$

(2) $$\begin{array}{l} \text{s.t.}\;\left\{\begin{array}{l} 0\leq B_{1}(t)\leq B_{1\max}\\ \frac{dM(\omega ,t)}{dt}=A\cdot M(\omega ,t)+b,\\ \;\frac{d\tilde{M}(\omega ,t)}{dt}=Ã\cdot \tilde{M}(\omega ,t)+b,\;\forall \omega \in \Omega ,\;\forall t\in (0,T_{\text{sat}}) \end{array}\right. \end{array}$$

The control, $B_{1}(t)$, describes the time‐dependent magnitude of the RF pulse. The first term in the cost function minimizes the energy of the RF pulse, regularized by α>0. The second and third terms minimize the difference to a target CW spectrum ($M_{\text{zdes}}$): first using a two‐pool Bloch‐McConnell simulation, where $M_{z}(\omega ,T_{\text{sat}})$ represents the longitudinal magnetization at frequency ω at the end of the saturation period $T_{\text{sat}}$; and secondly using the same simulation but considering only the water pool (${\tilde{M}}_{\text{zdes}}$ and $\tilde{M_{z}}$). The RF pulse is defined as a single pulse within a train of repeated pulses, separated by pauses of duration $t_{p}$. The simulation is discretized in time, t, using a step size of $\Delta t=10^{-4}$ s. Each RF pulse in the train has a duration of $t_{d}$, with a duty cycle of 90%. The gradient for optimization is computed over the entire pulse train and then averaged over the pulses to obtain a single gradient update for one pulse [31, 32]. The numerical optimization is based on a trust‐region semi‐smooth quasi‐Newton method [33]. Further details of the optimization can be found in [6] optimization time, convergence criteria, and sensitivity to start parameters are described in the supporting material.

The optimization was constrained to $T_{\text{sat}}=1$ s and a DC of 90%. To investigate different pulse saturation times, the pulse duration was set to $t_{d}=50$ and 100 ms with pulse pauses of $t_{p}=6$ and 12.5 ms. The regularization was set to α=1 and the weightings were set to σ=100. We iterate over p, starting at p=2 and incrementing successively as suggested by [33]. The optimization was initialized with a BPT and converged at p=4.

For the optimization, the simulated z‐spectra were discretized between 20 and −20 ppm with a step size of δω of 0.01 ppm, which results in $N_{\omega }=4001$ off‐resonant points. The water pool was set to resemble in vivo‐like tissue relaxation times at 3 T: $T_{1w}=1200$ ms and $T_{2w}=80$ ms. These values were chosen to be within the range of white and gray matter relaxation times at 3 T [34]. The solute pool was simulated with relaxation times $T_{1s}=1000$ ms and $T_{2s}=160$ ms and a fraction rate of $f_{s}=0.002$ at an off resonance of 3.5 ppm [35]. The exchange rate between the solute pool and the water pool was set to $k_{sw}=200$ s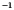. These pool parameters were chosen to strike a balance between labeling efficiency and direct saturation. The $B_{0}$ field was 3 T. The target spectrum was simulated with $T_{\text{sat}}=1$ s CW saturation pulse with an RF amplitude of $B_{1}=1$ µT.

### 2.2 Comparison of a Repeated Generalized OC Pulses Against CW and the FOPT in Simulation

The train of the generalized pulse is compared to the saturation of a CW pulse and the performance of the FOPT. This comparison is made for CEST pool offsets between 1 and 5 ppm, $B_{1RMS}$ values from 1 to 5 µT, exchange rates k=250, 1000, and 3000 s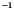, and a fraction rate $f_{s}=0.001$. The simulations are conducted with 90% DC and $T_{\text{sat}}=1$ s in two‐pool models at 3 T with a field inhomogeneity of $\Delta B_{0}=1$ ppm. The phases across the pulse trains were kept constant. The difference in performance is calculated with:

(3) $$\Delta_{\max}=\max\left|\frac{MTR_{\text{asym,ref}}-MTR_{\text{asym,sim}}}{MTR_{\text{asym,ref}}}\right|\times 100\%$$

where $MTR_{\text{asym,ref}}$ is the contrast metric obtained with the reference simulation (e.g., CW pulse) and $MTR_{\text{asym,sim}}$ is that from the simulation under comparison (e.g., generalized OC pulse train).

### 2.3 Generalization of OC Pulses Over Time and Magnetic Fields

To assess the influence of high‐frequency Fourier components in the OC pulse on the resulting CEST spectra, the OC pulse was low‐pass‐filtered using an 11th‐order Butterworth filter and compared to the unfiltered pulse. Simulations were conducted with a frequency sampling rate of δω=0.01 ppm over a range of −30 to 30 ppm, using a pulse train of 9 pulses, a duty cycle of 90%, and $B_{1}=1$ µT with $T_{1w}=1200$ ms, $T_{2w}=120$ ms, $T_{1s}=1000$ ms, and $T_{2s}=160$ ms.

To investigate the influence of doubling the single‐pulse saturation time and of the $B_{0}$ field strength, the filtered and unfiltered OC pulses were stretched by linear interpolation to 200 ms and simulated at 3 T and 7 T. Additionally, the filtered pulse was compressed to 75 ms and 50 ms for simulations at 3 T.

### 2.4 Pulseq Implementation

The OC pulses were incorporated into the Pulseq‐CEST framework with a newly added function makeOCPulse(fa_sat, ‘duration’, td, ‘useLowPass’, true_or_false). Here, the fa_sat is the flip angle over one pulse, td is the duration of a single pulse in ms, and true_or_false is an option for low‐pass filtering the OC pulses. The function generates a Pulseq‐CEST native pulse object that can be used arbitrarily. The default value for td is 100 ms, and for the useLowPass is false. More details about the implementation can be seen in the Supporting Information Section 11.

### 2.5 Phantom Measurements

The performance of OC saturation was compared to Gaussian, aSL, and Fermi saturation in phantom measurements on a Siemens Vida 3 T clinical scanner system (Siemens Healthineers, Erlangen, Germany) with a 20‐channel head coil. The sequences were implemented in Pulseq CEST. The phantom consists of four 50 mL Falcon tubes, each containing one of the following: 0.125 g nicotinamide (NA), 0.4 g Cr monohydrate, 5 mL Ultravist (300 mg/mL iopromide (IOP)), or 3 g sucrose as a model for hydroxyl (OH) groups (example image of the phantom can be seen in the Supporting Information Figure S13). The pH of the phantoms was stabilized at pH 7.0 with phosphate buffer. The relaxation times were reduced to approximately $T_{1}=1400$ ms and $T_{2}=120$ ms with MnCl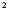. $B_{1}$ and $B_{0}$ maps were measured using the WASABI method [36].

Two experiments were performed:

1. For 50 ms pulses: duty cycles (DC) of 50% (10 pulses) and 90% (18 pulses) were applied.
2. For 100 ms pulses: duty cycles (DC) of 50% (5 pulses) and 90% (9 pulses) were applied.

All experiments were conducted with $B_{1RMS}$ values of 1, 1.5, and 2 µT. The spectra were measured at 45 frequency offsets, with higher sampling density around the CEST peaks at 1.2 ppm for OH groups, 1.7 ppm for Cr, 3.2 ppm for NA, and 4.1 ppm for IOP. $B_{0}$ inhomogeneities were corrected using $B_{0}$ maps from WASABI spectra, and the CEST effect was evaluated using the magnetization transfer ratio asymmetry ($MTR_{\text{asym}}$).

Furthermore, the nominal frequency spectra and the bandwidth of the single 100 ms Gaussian, Fermi, Block, and OC pulses were calculated using an FFT, and the FWHM was determined.

### 2.6 In Vivo Measurements

#### 2.6.1 Muscle Creatine Measurement

In vivo measurements were performed on a Siemens Vida 3 T clinical scanner (Siemens Healthineers, Erlangen, Germany) using an 18‐channel body coil on a generally healthy volunteer. The volunteer had a prior sports‐related muscle injury, which was incorporated as a variation in the scanned region. A $T_{2}$‐weighted transversal turbo spin echo image was acquired with the following scan parameters: $T_{R}=3000$ ms, $T_{E}=15$ ms, slice thickness = 5.5 mm, 30 slices, base resolution = 576 × 576, and FOV = 253 × 253 mm.

CEST images were generated using two different measurements, comparing a state‐of‐the‐art Gaussian saturation with an OC saturation. The Gaussian saturation was optimized to generate the highest Cr and PCr $MTR_{\text{asym}}$ at 3 T in calf muscle [37]. Gaussian saturation parameters: $B_{1RMS}=1.74$ µT, 90% DC, $t_{p}=50$ ms, 11 pulses, $T_{\text{sat}}=600$ ms. OC saturation was applied with the same $B_{1RMS}$ and DC but with 6 pulses of 100 ms each, resulting in a saturation time of $T_{\text{sat}}=650$ ms. The readout was a centric reordered 3 D gradient echo with parameters: $T_{R}$ = 4 ms, $T_{E}$ = 2.1 ms, slice thickness = 5 mm, 5 slices, base resolution = 128 × 128, FOV = 256 × 256 × 25 mm, flip angle α = 8°. $B_{1}$ and $B_{0}$ maps were measured using the WASABI method. The CEST measurement times were 11:23 min for Gaussian and 11:28 min for the OC‐based saturation.

The CEST spectra were generated with 101 offsets equidistant between −5 and 5 ppm. The spectra were $B_{0}$ corrected using the WASABI $B_{0}$ maps. The spectra were denoised using principal component analysis (PCA) using 14 principal components, and the number of components was determined with the median criterion as suggested by Breitling et al. [38]. CEST images were generated using the $MTR_{\text{asym}}$ peak at 1.9 ppm. The $MTR_{\text{asym}}$ images were denoised using an NLM filter with parameters: degree of smoothing = 0.015, comparison window = 3, and search window size = 31. The images were $B_{1}$ corrected using decorrelation of the CEST image with the $B_{1}$ map as suggested by Papageorgakis et al. [39]. For comparing the CEST spectra in vivo, 3 regions of interest (ROIs) were analyzed: one within the muscle lesion, one in a high‐SNR region with homogeneous $B_{1}$, and one in a region with high $B_{1}$ but low CEST SNR. Furthermore, SNR was calculated in a homogeneous ROI (124 pixels) within the vastus lateralis from the PCA denoised z‐spectra.

#### 2.6.2 Brain APT Measurement

Brain APT measurements were performed on the same scanner using a 20‐channel head coil on a healthy volunteer (female, 32 years). The same 3D gradient echo readout sequence was used as described above. CEST saturation was based on the consensus paper protocol B2 for 3 T APT measurements [40]. Gaussian saturation parameters followed the consensus recommendations: 100 ms Gaussian pulses applied 18 times with 10 ms pauses, resulting in a 91% DC, $B_{1RMS}$ = 1 µT, and $T_{\text{sat}}=2$ s to enable Lorentzian fitting. Measurement time per protocol was 9:28 min. OC saturation was applied with identical parameters but using the OC pulse shape.

CEST spectra were acquired using offsets recommended for Lorentzian fitting [41] with 10 frequency points between 3 and 4 ppm, in total 59 offsets. Spectra were $B_{0}$ corrected using WASABI $B_{0}$ maps. APT contrast was estimated by subtracting the background using 2‐pool Lorentzian fitting for water and MT, with Lorentzian difference analysis for APT contrast quantification. The measurement spectra were denoised using PCA.

For comparison between Gaussian and OC saturation, 3 ROIs were analyzed, and the mean of the spectra with maximum intensity at 3.5 ppm was used as the comparison metric.

Written informed consent was obtained from the volunteers, and the study was approved by the local ethics committee.

## 3 Results

### 3.1 Comparison of a Repeated Generalized OC Pulses Against CW and the FOPT in Simulation

**FIGURE 1:**
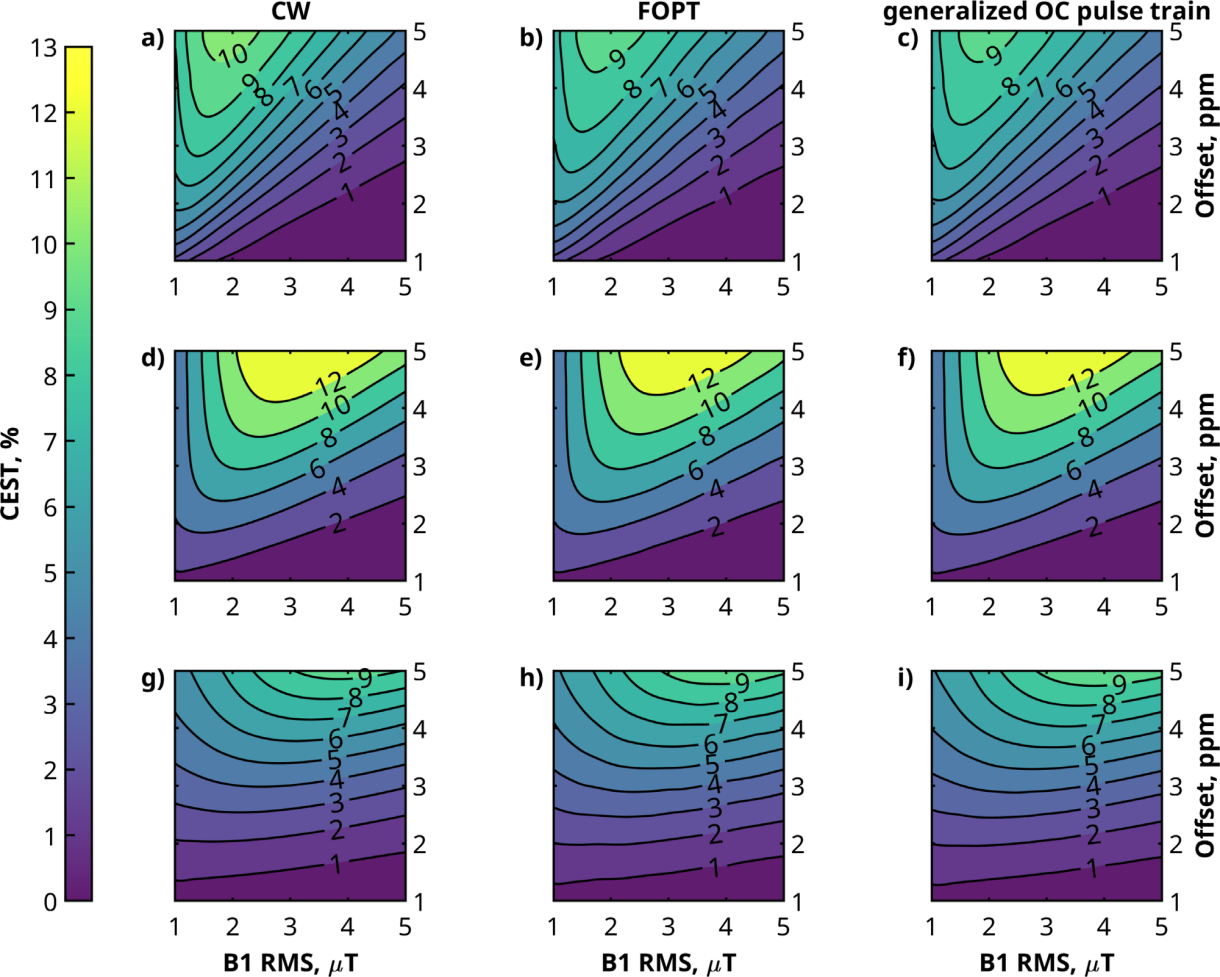
CEST effect generated by the theoretically optimal CW saturation (a, d, g) in comparison to the FOPT (b, e, h) [6] and the generalized OC pulse (c, f, i). All pulses were tested in a two‐pool simulation with $T_{\text{sat}}=1$ s, for clinically relevant $B_{1RMS}$ values and off‐resonances of the CEST pool. The OC trains have a DC of 90%. Simulations were performed for different exchange rates: 250 s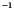 (a, b, c), 1000 s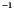 (d, e, f), and 3000 s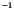 (g, h, i). For all cases, a $B_{0}$ inhomogeneity of 1 ppm was used.

Figure 1 presents contour plots of the simulated CEST effect for CW, the FOPT, and the proposed generalized OC saturation pulse train across $B_{1RMS}$ values (1–5 µT) and CEST pool offsets (1–5 ppm). Panels (a–c) display results for k=250 s^−1^, (d–f) for k=1000 s^−1^, and (g–i) for k=3000 s^−1^. All saturation strategies show a similar well‐known trend: the CEST effect increases with offset and peaks at a specific $B_{1RMS}$, depending on the exchange rate. For k=250 s^−1^, the maximum occurs at 2 µT, for k=1000 s^−1^ at higher $B_{1RMS}$ around 2–4 µT, and for k=3000 s^−1^ between 3 and 5 µT.

Near the peak CEST effect, CW saturation generates a slightly larger effect than both the FOPT and generalized OC saturation strategies. For k=250 s^−1^, the highest difference are 9.3% (FOPT) and 10.4% (generalized OC); for k=1000 s^−1^, 4.1% and 4.4%; for k=3000 s^−1^ both OC saturations are slightly higher than the CW saturation at the maximum. The $B_{0}$ inhomogeneities of 1 ppm lead to no visible artifacts in the performances of the OC strategies.

### 3.2 Generalization of OC Pulses Over Time and Magnetic Fields

**FIGURE 2:**
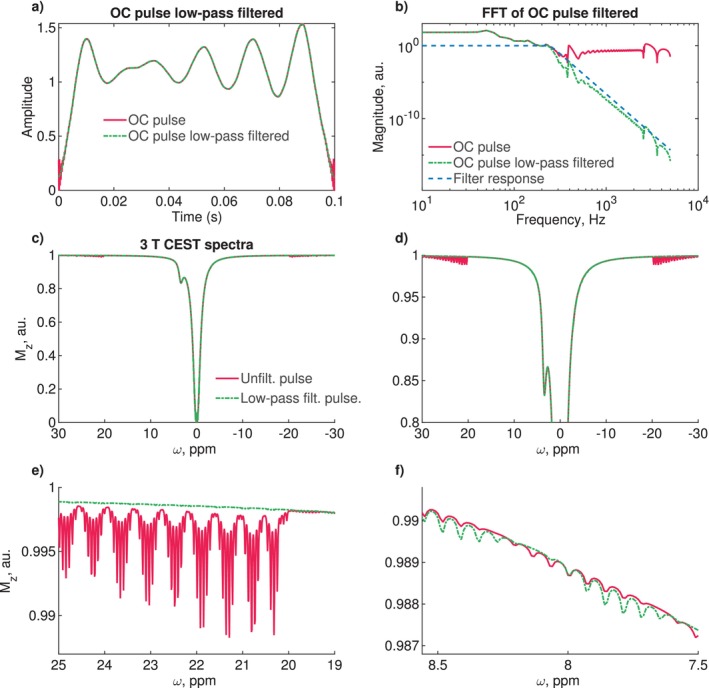
Low‐pass filtering of the OC pulse: (a) The original pulse from the optimization and the low‐pass‐filtered pulse. (b) The frequency spectrum of the original and low‐pass‐filtered OC pulse, along with the response of the low‐pass filter. (c) Simulated CEST spectrum at 3 T with very fine frequency sampling of ≈1.3 Hz (0.01 ppm). (d) The same spectrum, zoomed in to highlight sidebands at offsets > 2.6 kHz (20 ppm at 3 T). (e) Further zoom into the sideband structure. (f) Zoom into sidebands between 0.96 and 1.09 kHz (7.5 and 8.5 ppm at 3 T).

Figure 2a shows the optimized 100 ms OC pulse as obtained from the optimization process, alongside its low‐pass‐filtered version. The corresponding FFT of the original and filtered pulses, and the low‐pass filter response, are displayed in (b). The low‐pass filter suppresses frequencies above 250 Hz, preserving the overall pulse shape while smoothing oscillations, particularly at the pulse's beginning and end.

The impact of the low‐pass‐filtered pulse on the simulated CEST spectrum at 3 T is shown in Figure 2c–f. For the unfiltered OC pulse, prominent sidebands are observed at offsets outside ±2.6 kHz (±20 ppm at 3 T). The low‐pass‐filtered pulse generates a smoother spectrum in these regions.

OC saturation also introduces sidebands inside the optimized frequency range, as exemplified in (f), with a maximum amplitude in the order of 1e‐4 of the water peak. The low‐pass‐filtered pulse introduces wiggles with slightly higher amplitude in the CEST spectrum.

**FIGURE 3:**
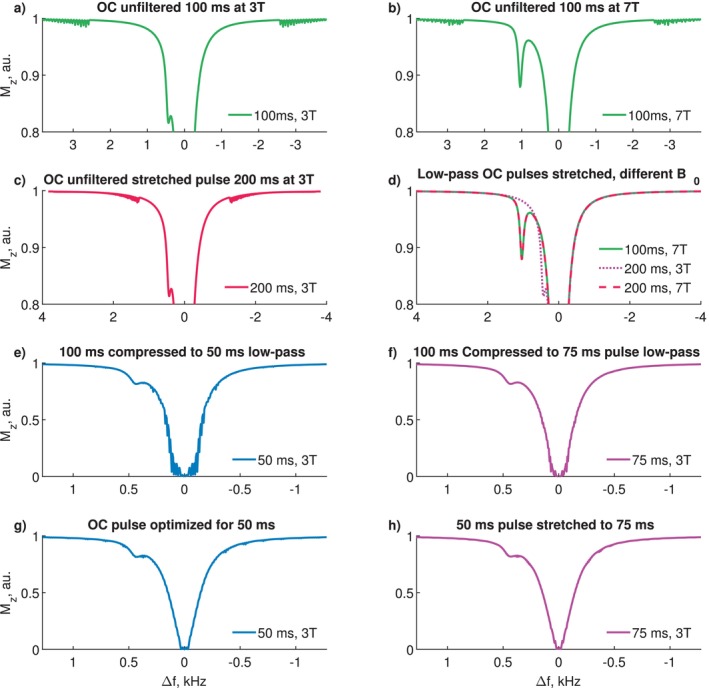
Simulations for filtered and unfiltered OC pulses stretched and compressed in time at different $B_{0}$ field strengths: (a) Unfiltered 100 ms pulse simulated at 3 T with sampling of dω=1.3 Hz (0.01 ppm), showing sidebands at offsets >2.6 kHz (>20 ppm). (b) The same pulse was simulated at 7 T with sidebands at offsets >3 kHz (>10 ppm). (c) The 100 ms pulse is interpolated in time and stretched to 200 ms, showing sidebands at offsets >3 kHz (>10 ppm). (d) Low‐pass‐filtered OC pulse simulated with 100 ms at 7 T and the filtered OC pulse stretched to 200 ms, simulated at 3 T and 7 T. (e) Simulation of the OC 100 ms pulse compressed to 75 ms, showing oscillations in the water peak. (f) Simulation of the OC 100 ms pulse compressed to 50 ms, showing stronger oscillations in the water peak.

Figure 3a,b shows simulated CEST spectra with 100 ms unfiltered OC pulses. (a) is at 3 T over ±3.8 kHz (±30 ppm), and (b) is at 7 T. Sidebands appear outside the optimized range (±2.6 kHz, ±20 ppm). Interpolating the 100 ms unfiltered OC pulse to 200 ms shifts the sidebands from outside ±2.6 kHz (±20 ppm) to within ±1.3 kHz (±10 ppm), as shown in (c).

Figure 3d shows simulated CEST spectra for low‐pass‐filtered OC saturation pulses under various conditions: 100 ms at 7 T, 200 ms at 3 T, 200 ms at 7 T. Sidebands observed with the unfiltered pulse were not detectable in the low‐pass‐filtered version across all stretched pulse durations and $B_{0}$ field strengths.

Figure 3e,f shows CEST spectra simulated with a 100 ms pulse compressed and resampled to 75 ms and 50 ms, respectively. Figure (g, h) depict simulations of an OC pulse optimized for 50 ms and the same pulse stretched to 75 ms. Resampling the 100 ms pulse to 75 ms introduces noticeable oscillations in the water peak, which become more pronounced when further reduced to 50 ms. In contrast, the pulse optimized for 50 ms exhibits fewer oscillations, even when stretched to 75 ms. The OC pulse optimized for 50 ms and its interpolation to 75 ms exhibits minor sidebands distributed throughout the spectra.

### 3.3 Phantom Measurements

**FIGURE 4:**
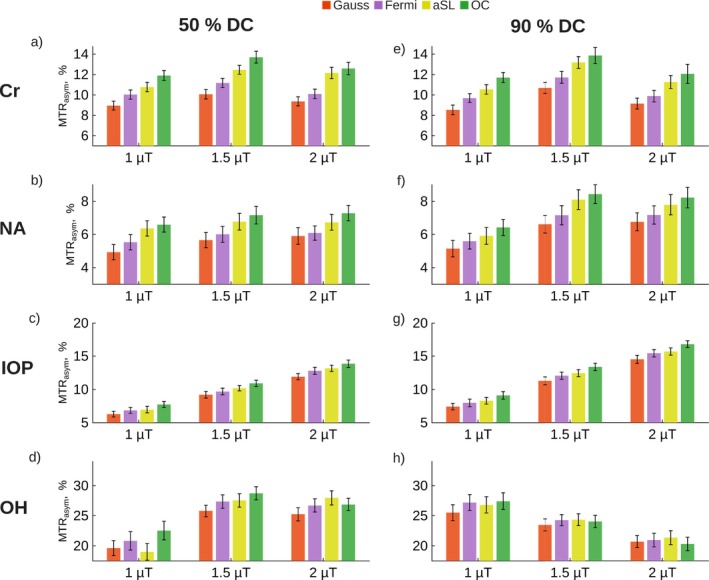
Phantom measurements comparing the performance of Gaussian, Fermi, adiabatic Spin Lock (aSL), and OC saturation pulses. All pulses were applied for 100 ms with a total saturation time of 1 s, using 50% duty cycle (DC) in (a–d) and 90% DC in (e–h). Saturation was performed at $B_{1}$ RMS of 1, 1.5, and 2 µT. The phantom consisted of four Falcon tubes, each containing one of the following: Cr, NA, IOP, and sucrose (OH).

Figure 4 compares the performance of four saturation pulses (Gaussian, Fermi, aSL, and OC) under varying conditions of duty cycle (50% and 90%) and $B_{1RMS}$ of (1, 1.5, and 2 µT). Across all measured regimes, the $MTR_{\text{asym}}$ values varied depending on the pulse and the experimental conditions. Generally, 90% DC generated higher contrast than 50%. In the Cr and NA phantom, the highest contrast was generated by 1.5 µT $B_{1RMS}$ and 90% DC across all saturation strategies. In the IOP phantom, the generated CEST contrasts increased with higher $B_{1RMS}$ and DC values.

Differences in $MTR_{\text{asym}}$ values between the OC pulse and the Gaussian pulse ranged from 15.7% to 37.1% across all offsets and conditions. Differences between the OC and Fermi pulses ranged from 8.3% to 24.7%, while differences between the OC and aSL pulses were between 3.5% and 11.2%. The highest differences between the OC pulse and other saturation pulses were observed in the Cr measurement, with values ranging from 29.7% to 37.1% compared to the Gaussian pulse, 18.4% to 24.7% compared to the Fermi pulse, and 3.5% to 11.0% compared to the aSL pulse. These differences were followed by those in the NA measurement, which ranged from 21.6% to 33.7% for the Gaussian pulse, 14.5% to 19.6% for the Fermi pulse, and 3.7% to 8.5% for the aSL pulse. The lowest differences were observed in the IOP measurement, with ranges of 15.7% to 22.9% for the Gaussian pulse, 8.3% to 14.0% for the Fermi pulse, and 5.2% to 11.2% for the aSL pulse.

In Figure S1 (Supporting Information), the same phantom and parameters are compared as in Figure 4 but for 50 ms pulses. Across all measured regimes, the $MTR_{\text{asym}}$ values followed similar trends to the 100 ms pulse durations, with higher contrast generally observed at 90% DC compared to 50%. In the Cr and NA phantoms, higher $B_{1RMS}$ and DC yielded greater contrast, while in the IOP phantom, this trend was consistent across all conditions.

The $MTR_{\text{asym}}$ differences between the OC pulse and the Gaussian pulse ranged from 8.1% to 29.0%, and between the OC and Fermi pulses from 3.1% to 17.9%. Differences between the OC and aSL pulses ranged from 10.2% to 33.4%, with the highest differences observed in the Cr measurement, followed by the NA and IOP measurements. In the Cr measurement, the differences ranged from 21.2% to 29.0% for the Gaussian pulse, 10.1% to 17.9% for the Fermi pulse, and 14.3% to 26.6% for the aSL pulse. For the NA measurement, the differences ranged from 13.5% to 19.2% for the Gaussian pulse, 5.5% to 13.6% for the Fermi pulse, and 10.2% to 26.9% for the aSL pulse. The IOP measurement showed the lowest differences, ranging from 8.1% to 25.0% for the Gaussian pulse, 3.1% to 16.0% for the Fermi pulse, and 11.2% to 33.4% for the aSL pulse.

The frequency spectra of single 100 ms Gaussian, Fermi, Block, and OC pulses show bandwidths of 20 Hz, 17 Hz, 12 Hz, and 13 Hz, respectively (see supporting material (Figure S12, Supporting Information)).

### 3.4 In Vivo Measurement

#### 3.4.1 Muscle Creatine Measurement

**FIGURE 5:**
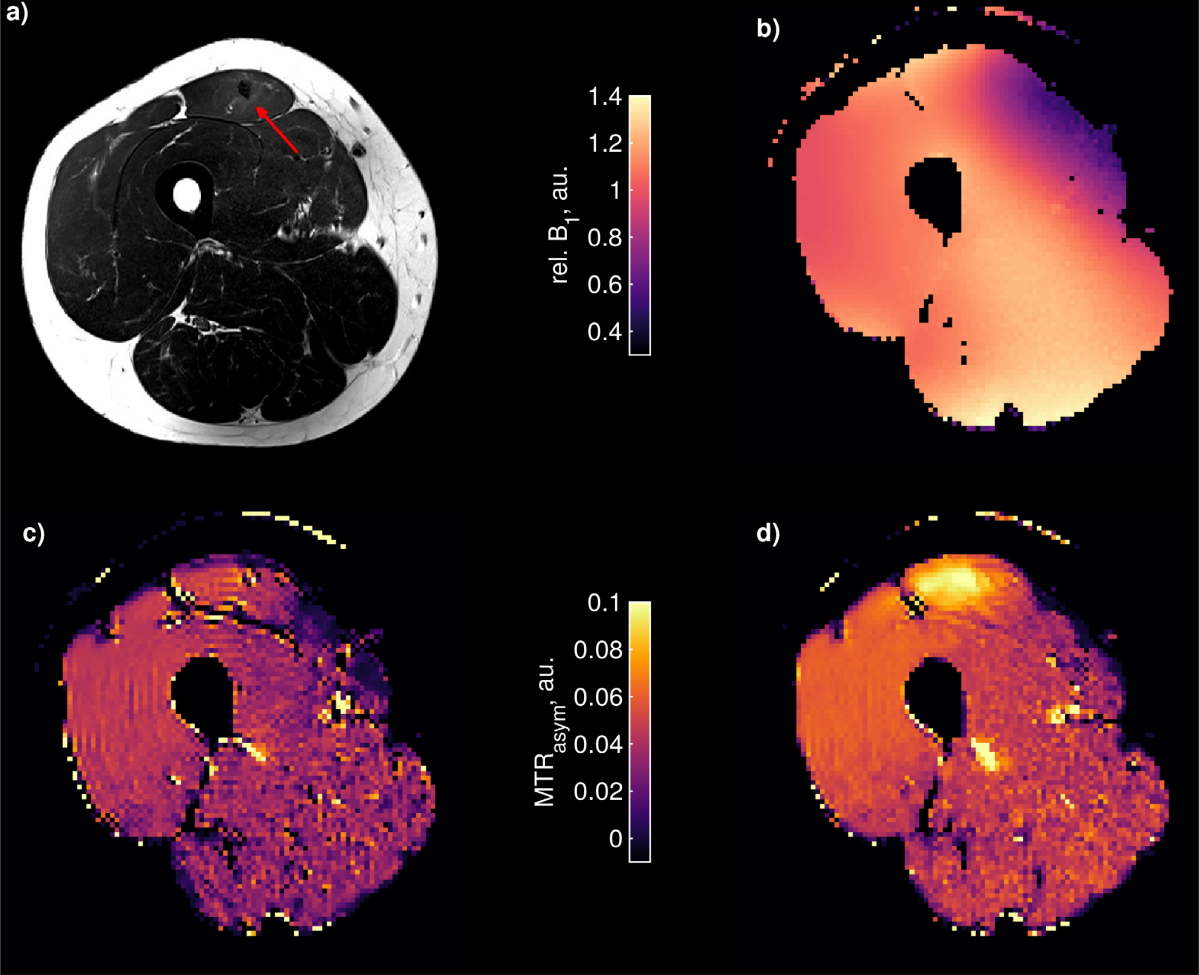
Comparison of Gaussian versus OC saturation for creatine CEST imaging in thigh muscle with an injury in the rectus femoris: (a) $T_{2}$‐weighted image with increased values see red arrow, (b) relative $B_{1}$ map, (c) Gaussian saturation protocol with 11 pulses of 50 ms each and a duty cycle (DC) of 90%, with a $B_{1,\text{RMS}}$ of 1.74 µT, and (d) generalized OC saturation with 6 pulses of 100 ms each, also with a 90% duty cycle, and a $B_{1,\text{RMS}}$ of 1.74 µT. The measurement spectra were denoised using PCA, while the creatine CEST maps were denoised using NLM. The creatine CEST maps were $B_{1}$ corrected using decorrelation of the CEST and $B_{1}$ map as proposed by [39]. Some hyperintensities are apparent in the CEST images. These are flow‐related artifacts due to blood vessels.

Figure 5 shows a transverse section through the right thigh. (a) displays a $T_{2}$‐weighted image highlighting the injury in the rectus femoris, with increased $T_{2}$ signal intensity (red arrow). (b) shows a $B_{1}$ map, with $B_{1}$ values ranging from 0.5 to 1.4 µT. (c, d) show Cr and PCr $MTR_{\text{asym}}$ CEST images acquired with the Gaussian saturation protocol and the generalized OC saturation. Elevated Cr and PCr contrast is visible in the rectus femoris and parts of the vastus intermedius in both Gaussian and OC images. The OC images exhibit higher contrast and more uniform contrast distribution, with fewer hyper‐intense regions and higher contrast to noise compared to the Gaussian images.

**FIGURE 6:**
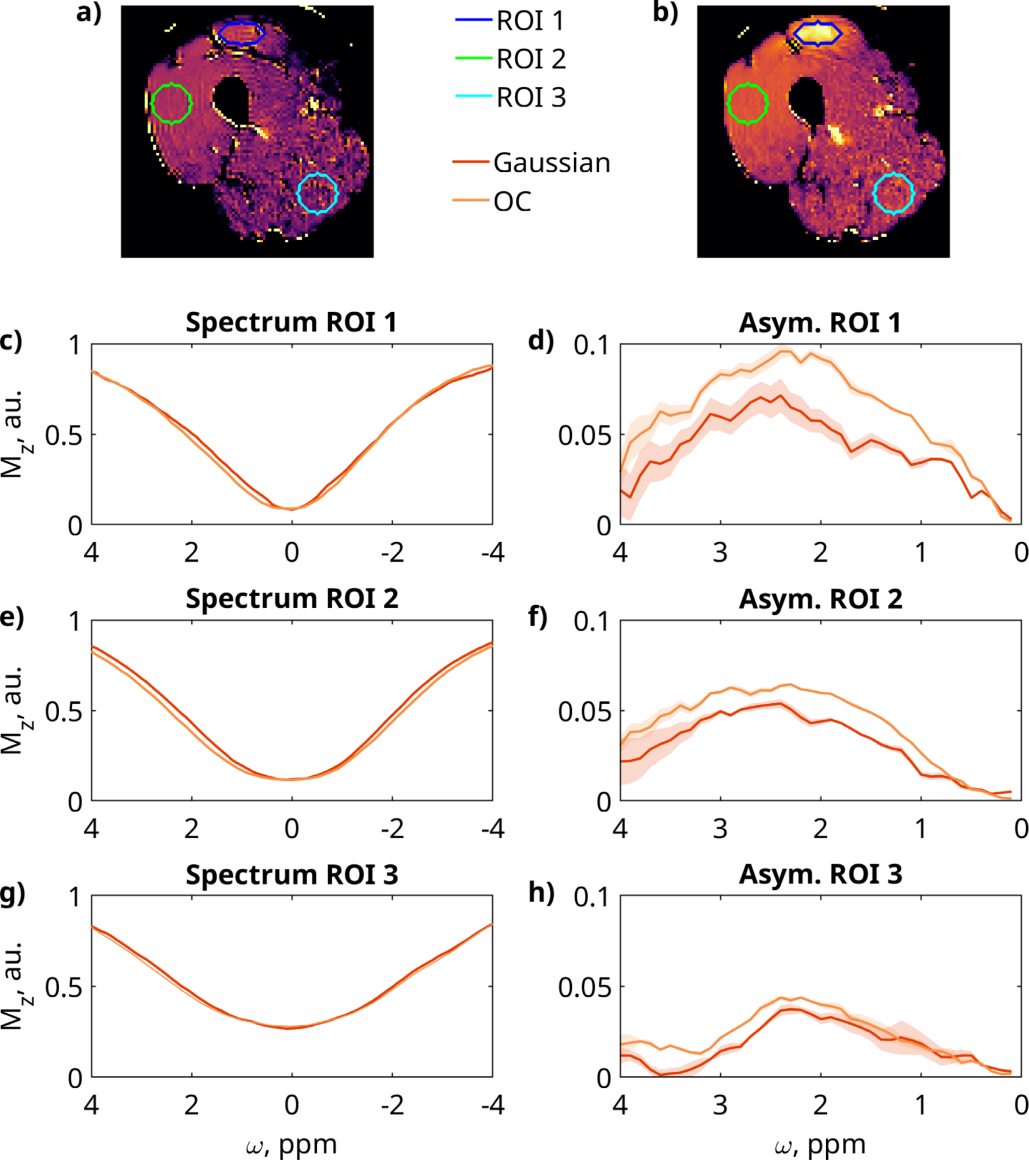
Comparing in vivo CEST spectra in different ROIs: (a) Creatine CEST image generated with Gaussian saturation with ROIs. (b) Creatine CEST image generated with OC saturation with ROIs. CEST spectra and $MTR_{\text{asym}}$ with standard deviation in the lesion region of the rectus femoris, (c,d) in the vastus lateralis, and (g,h) in a noisy, low $B_{1}$ region.

Figure 6 shows the mean value of $MTR_{\text{asym}}$ from ROIs in three regions of the CEST images generated with (a) the Gaussian saturation and (b) the OC saturation. (c, e, g) display the full CEST spectra in the rectus femoris lesion area ROI, while (d, f, h) highlight the $MTR_{\text{asym}}$ peaks.

The mean $MTR_{\text{asym}}$ values and standard deviations for each ROI are as follows: For ROI 1, OC saturation was 0.090±0.003 and Gaussian saturation was 0.053±0.006, resulting in 70.4% higher mean and 50.5% lower standard deviation for OC saturation. For ROI 2, OC saturation was 0.0592±0.0006 and Gaussian saturation was 0.0448±0.0011, leading to 32.1% higher mean and 47.3% lower standard deviation for OC saturation. For ROI 3, OC saturation was 0.0391±0.0022 and Gaussian saturation was 0.0330±0.0028, yielding 18.6% higher mean and 20.3% lower standard deviation for OC saturation. Across all ROIs, the OC saturation consistently yields higher $MTR_{\text{asym}}$ peak values compared to the Gaussian saturation. SNR calculation in the vastus lateralis yielded values of 4.16 for Gaussian saturation and 6.68 for OC saturation. The ROI used for the calculation can be seen in Figure S21 of the Supporting Information.

#### 3.4.2 Brain APT Measurement

**FIGURE 7:**
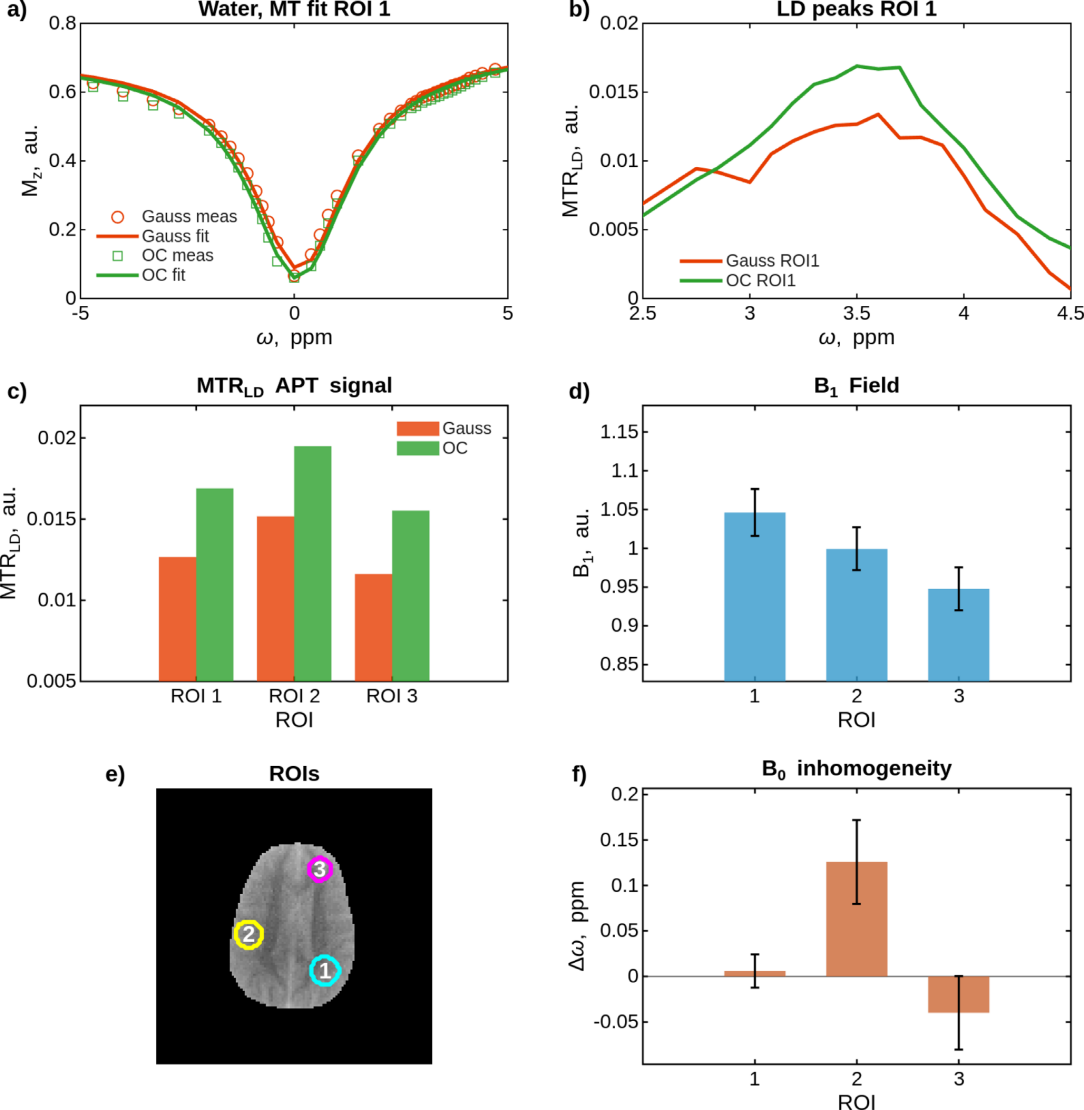
In vivo brain APT‐CEST imaging at 3T with 90% DC, $T_{\text{sat}}=2$ s, $B_{1RMS}=1$ µT for $t_{p}=100$ ms Gaussian and OC saturation pulses. (a) Z‐spectra fitted with two‐pool Lorentzian model (water and MT pools), (b) Lorentzian difference spectra after background subtraction, (c) $MTR_{LD}$ values at 3.5 ppm across three ROIs, (d) $B_{1}$ field distribution and standard deviation within ROIs, (e) $T_{1}$‐weighted image showing ROI locations, (f) $B_{0}$ distribution within ROIs.

Figure 7 shows the results of the APT measurement in the brain using Lorentzian fitting. (a) displays the measurement points in ROI 1 with the fitted Lorentzian background for Gaussian and OC saturation, (b) shows the Lorentzian difference MTR(LD) in ROI 1. (c) depicts the mean values of the $MTR_{LD}$ in ROI 1, 2, and 3 which are shown in (e). The $B_{1}$ and $B_{0}$ distributions (d,f) in the ROIs were: ROI 1: (1.05±0.03) µT and (0.01±0.02) ppm, ROI 2: (1.00±0.03) µT and (0.13±0.05) ppm, ROI 3: (0.95±0.03) µT and (−0.04±0.04) ppm, respectively. The OC APT contrast was 33.1%, 28.3%, and 33.6% higher in ROIs 1, 2, and 3, respectively. The parameter maps for water, MT, and APT can be seen in the Supporting Information Figure S20.

## 4 Discussion

In previous work, we introduced an OC saturation pulse train for enhanced and robust contrast in CEST MRI on clinical scanners [6]. This pulse train was optimized as a whole for a specific saturation duration with a fixed duty cycle. Changing these parameters requires a new optimization, which limits the flexibility for experimental use.

Here, we propose a generalized OC pulse that is optimized as a single unit and can be repeated to form a pulse train, enabling adaptation to arbitrary DC, $T_{\text{sat}}$, $t_{d}>50$ ms, $B_{1}$ levels, offsets, exchange rates, and all frequency ranges, making it easy to use for all $B_{0}$ fields. We demonstrate its excellent performance across all investigated regimes by comparing it to CW saturation in simulations, to state‐of‐the‐art saturation strategies in phantom experiments across various CEST agents, and to Gaussian saturation optimized for Cr and PCr CEST contrast in an in vivo example. The proposed pulses are integrated into the Pulseq‐CEST framework, providing an easily accessible alternative to conventional saturation pulses.

The optimization of RF saturation pulses for CEST MRI has also been explored by other groups. Yoshimaru et al. [17] applied a genetic algorithm using smooth basis functions to optimize pulsed‐CEST saturation, ensuring a controlled spectral profile but inherently limiting the achievable contrast due to constraints in the solution space. More recently, Mohanta et al. introduced a 100 ms free‐form optimized pulse for 11.7 T and 99% DC, optimizing for a single spectral offset but without explicitly addressing spectral properties for imaging. In this work, we introduce a generalized OC pulse that optimizes across the full spectral range, ensuring high and robust contrast while maintaining spectral stability and mitigating sideband artifacts and Rabi oscillations across various physical and measurement parameters.

### 4.1 Comparison to CW and FOPT in Simulation

Figure 1 demonstrates that the proposed OC pulse generates CEST contrast comparable to the published pulse across all tested conditions. This includes offsets between 1 and 5 ppm, $B_{1RMS}$ values from 1 to 5 µT, and exchange rates of 250, 1000, and 3000 s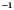, covering most clinically relevant CEST agents. Near the peak CEST effect, CW saturation produces about 10% higher contrast than OC strategies at k=250 s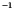 and about 4% higher at k=1000 s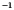. These deviations are mainly due to the shorter effective saturation time for a duty cycle of 90% and the same $T_{\text{sat}}$. However, at k=3000 s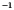, both OC and FOPT pulses slightly exceed CW saturation due to a slightly lower spillover. Moreover, the simulated $B_{0}$ field inhomogeneity of 1 ppm demonstrates the robustness of the OC saturation. The $B_{0}$ robustness is indirectly enforced in the simulation by maintaining a constant phase offset between pulses. For a more detailed explanation, see [6]. Further simulations with different tissue parameters of OC pulses in comparison to CW can be seen in [6].

### 4.2 Generalization Over Frequency, $B_{0}$ and $t_{d}$

To ensure a stable spectrum generated by OC pulses, the simulated spectrum is optimized to resemble a CW spectrum with 4001 equidistant frequency components. The high‐frequency sampling (0.01 ppm) is necessary to show sideband artifacts [2] and consequently suppress sidebands and Rabi oscillations. However, this stability is only ensured within a limited frequency range, that is, ±2.6 kHz (20 ppm at 3 T). Beyond this range, saturation introduces small artifacts (Figure 2c,d). Theoretically, wider ranges are possible, but ±20 ppm cover more than the relevant frequency range and avoid unnecessarily inflating the optimization problem.

The artifacts outside the optimized spectral range could be mitigated by restricting high frequencies in the pulse via low‐pass filtering. Suppressing frequencies above 250 Hz in the OC pulse resulted in a smoother spectrum outside the optimized frequency range (<−2.6 kHz and >2.6 kHz). Yet, OC saturation introduces slight oscillations in the spectrum (Figure 2f), which slightly increase with low‐pass filtering. These oscillations, on the order of $10^{-4}$ of the water signal, are negligible in measurements. Thus, low‐pass filtering improves the pulse behavior beyond the optimized frequency range.

The relevant frequency range for CEST experiments depends on the $B_{0}$ field strength. When the unfiltered pulse is simulated at 7 T at ±20 ppm (or ±6.0 kHz), wiggles appear at approximately 8.6 ppm (2.6 kHz) (see Figure 3b). A similar effect is observed when the OC pulse is stretched to 200 ms at 3 T (Figure 3c), where the wiggles appear at 1.3 kHz (10 ppm). Figure 3d shows that low‐pass filtering eliminates these artifacts in all cases, including the combination of 200 ms at 7 T. Therefore, low‐pass filtering generalizes OC saturation across different $B_{0}$ field strengths and for pulse durations $t_{d}>100$ ms.

Compressing a 100 ms pulse to $t_{d}<100$ ms is less straightforward. Figure 3e,f shows simulations for 50 ms and 75 ms pulse durations. At 75 ms, artifacts begin to appear within ±160 Hz (1.25 ppm) and become more pronounced at 50 ms, where they can be observed in the range of ±320 Hz (2.5 ppm) at 3 T.

To minimize these artifacts for short OC pulses, a dedicated pulse was optimized for 50 ms. Its performance is shown in Figure 3g, along with its stretched version at 75 ms. The 50‐ms‐optimized pulse exhibits significantly fewer artifacts than the compressed 100 ms pulse. Furthermore, stretching this optimized 50 ms pulse to 75 ms reduces wiggles near the water peak compared to simply compressing a 100 ms pulse to 75 ms. However, stretching short pulses too far, for example, from 50 ms to 100 ms, degrades performance compared to pulses optimized directly for 100 ms.

All shaped CEST pulses generally introduce some amount of Rabi oscillations and sidebands into the spectrum. Rabi oscillations depend on the adiabatic properties of the pulse shape, and slowly varying RF pulses show fewer oscillations. The origin of sidebands is more complex; they emerge when some pulse shape‐dependent resonance condition is fulfilled at some off‐resonant frequencies [2].

### 4.3 Phantom Measurements

To assess the performance of generalized OC pulses in phantom CEST measurements, we selected four agents covering a broad range of chemical shifts and exchange rates (1.2–4.1 ppm). Fast‐exchanging OH groups at 1.2 ppm and slow‐exchanging Cr at 1.7 ppm, which is close to water, are more susceptible to spillover, artifacts, and Rabi oscillations. In contrast, NA (3.2 ppm) and IOP (4.1 ppm) are farther from water, reducing spillover effects.

Across all investigated $B_{1RMS}$ levels, offsets, and exchange rates, OC pulses achieved the highest saturation efficiency for 50 ms and 100 ms durations with duty cycles of 50% and 90%. The only exception was OH measurements, where aSL led to higher saturation at higher $B_{1RMS}$ levels due to its lower direct water saturation. However, OC saturation produced higher overall contrast for OH exchange, even at lower $B_{1RMS}$ levels.

For Cr, NA, and IOP, 100 ms pulses outperformed 50 ms, while OH groups responded better to 50 ms. aSL was the exception, performing markedly worse at 50 ms across all regimes. BPT saturation data were excluded due to their sensitivity to $B_{0}$ inhomogeneities (up to 0.1 ppm). Additional simulations for the performance of the different pulses over varying $B_{1RMS}$ values can be seen in Figure S16 in the Supporting Information.

### 4.4 In Vivo Measurements

#### 4.4.1 Muscle Creatine Measurement

Muscle was chosen for in vivo experiments due to its pronounced Cr and PCr CEST peaks at 3 T. Measurements were performed in the thigh muscle of a generally healthy volunteer (Male, 28 years), with a prior sports‐related muscle injury. The experiment compared the generalized OC pulse to a previously optimized Gaussian saturation for Cr and PCr imaging [37]. The OC pulse train was configured to replicate the Gaussian train as closely as possible. To meet the 90% duty cycle, six OC pulses with longer pauses were used, making the pulse train 50 ms longer compared to the Gaussian train.

OC saturation provided higher and more homogeneous CEST contrast at 1.9 ppm, with less noise and much higher saturation in low $B_{1}$ regions (≈40% lower) in comparison to Gaussian saturation. In ROI 3, where the relative $B_{1}$ was approximately 30% higher, OC saturation yielded a 19% increase in contrast.

Due to coil inhomogeneities and artifacts at tissue boundaries, it is difficult to specify the SNR for different saturation pulses exactly. However, Figure 6 shows that the mean $MTR_{\text{asym}}$ value for the OC pulse is higher at all $B_{1}$ levels in the three ROIs, and that the associated variance for the OC pulse is lower. In contrast to the vastus lateralis, which has the fewest artifacts and homogeneous $B_{1}$ (rel. $B_{1}\approx 1.0$), the SNR is 4.16 for Gaussian saturation and 6.68 for OC saturation, representing a 61% improvement with OC saturation. Further discussion on the SNR calculation is given in Section 13 of the Supporting Information

Interestingly, the contrast was markedly increased in the region with the muscle lesion (ROI1). To the best of our knowledge, this is the first detection of elevated Cr contrast in muscle tissue adjacent to a rupture detected using CEST imaging.

One possible explanation is that Cr leakage from ruptured muscle cells increases its extracellular concentration, where it has greater exposure to water, leading to higher contrast. Kogan et al. demonstrated that Cr CEST imaging, in contrast to magnetic resonance spectroscopy, is a suitable method for detecting free Cr in muscle [42, 43], supporting this hypothesis. Another possibility is that local Cr metabolism is altered in response to muscle injury.

#### 4.4.2 Brain APT Measurement

The APT measurement was chosen to contrast the muscle measurement, as the brain provides a controlled environment with less field inhomogeneities where APT contrast is well‐established and Lorentzian fitting can reliably separate individual CEST pools. Unlike the muscle measurement where only $MTR_{\text{asym}}$ analysis was feasible due to the high $B_{1RMS}$ (1.74 µT) and low $T_{2}$ conditions that produce excessively broad spectra unsuitable for Lorentzian fitting, the brain measurement conditions ($B_{1RMS}$ = 1 µT, broad frequency sampling) enabled robust Lorentzian fitting of water and MT for isolated APT quantification.

Lorentzian difference analysis isolates the APT pool without NOE interference, enabling direct comparison of labeling efficiency between pulse shapes. In standard $MTR_{\text{asym}}$ analysis, a specific saturation can cause increased labeling of both the APT and NOE pools, which then cancel each other out in the asymmetry calculation. Further explanation and in vivo APT $MTR_{\text{asym}}$ measurements for different $B_{1}$ levels can be seen in the Supporting Information Section 14, Figure S22.

The OC saturation demonstrated consistently ≈30% higher APT contrast compared to the consensus Gaussian protocol across all ROIs, which showed minimal $B_{0}$ and $B_{1}$ field variations. This improvement exceeds NA phantom measurements, confirming that OC saturation benefits can be extended to physiologically relevant conditions.

### 4.5 Single‐Pulse Optimization Trade‐Off

Transitioning from a pulse train where each pulse is different to individually optimized single pulses significantly increases the versatility of the approach. A key advantage of this design is that each pulse prepares the magnetization completely independent of the previous pulse. This independence enables interleaved measurements, where the spectrum can be acquired after each pulse. This technique allows for studying magnetization dynamics in greater detail. Potential applications include motion correction, exchange rate estimation, and integrating $T_{1}$ mapping within the same experiment. This effect is demonstrated in Figure S2 (Supporting Information), where simulations show how a smooth spectrum forms after each saturation pulse.

Optimizing a single 100 ms pulse introduced slight artifacts in the water spectrum between −1 and 1 ppm at 3 T, which could not be fully suppressed within a single‐pulse design. These features are only apparent at high spectral resolution (Δω=0.01ppm) and are also commonly observed in other saturation strategies [2]. An alternative cost functional allowed for solutions where either two alternating pulses or a pulse train terminating with a second, distinct pulse effectively mitigated these wiggles, yielding a spectrum comparable to the FOPT [44]. These strategies, along with the modified cost functional, are detailed in the Supporting Information (Figure S3).

Minor residual oscillations in the single‐pulse design typically do not impact the measured CEST effects. RF spoiling after each pulse further suppresses these artifacts, as shown in simulations and phantom data (see Supporting Information Figures S4, S5). Other RF pulses, for example, Gaussian and Fermi, also introduce spectral artifacts (see Supporting Information Figure S6). Given the complexity of the two‐pulse approach, the simpler single‐pulse design remains preferable despite minor imperfections. Nonetheless, we propose an alternative method to further reduce spectral artifacts.

### 4.6 Choice of the Target Spectrum

Many seminal publications are based on CW saturation, or refer to it as an objective to be achieved [16, 45, 46]. Technical adjustments to specific whole‐body scanners that adapt the RF system for CW saturation also emphasize this point [40]. For multi‐pool systems at 7 T and low exchange rates, we (M. Z.) found that Gaussian saturation pulses of a suitable amplitude can achieve greater saturation than CW saturation. A similar effect was described previously by van Zijl et al. [47], who found that higher fields reduced NOE contrast due to increased selectivity. Simulations show that this effect occurs within a specific $B_{1RMS}$ exchange rate window (see Supporting Information Figures S7–S9 of the Supporting Information). For this multi‐pool system, an OC pulse was subsequently optimized Figure S10 and S11 to demonstrate the flexibility of the pulse design.

In single‐pool optimizations, OC phase variation was allowed, but the optimal solution consistently yielded zero phase. In contrast, multi‐pool (5 pool) OC optimization produced pulses with a phase sweep (see Figure S10, Supporting Information), outperforming CW saturation at low exchange rates (see Figure S11). The target spectrum was based on short Gaussian pulses, but the optimal target in the multi‐pool case remains unclear. Future work should explore improved targets and the role of frequency sweeps in enhancing CEST contrast in multi‐pool peaks.

This work is a methodological development for optimizing the design of saturation pulses, and with the change of the target and more or less degrees of freedom for the RF pulse, it is a flexible framework for various challenges.

## 5 Conclusion

With this work, we demonstrate that previously designed OC saturation trains can be largely generalized using a single unit that can be flexibly applied and demonstrates strong performance across all investigated duty cycles, exchange rates, offsets, $B_{1RMS}$ values, and $B_{0}$ field strengths. The performance of the OC pulses has been validated through simulations against CW saturation and phantom measurements against state‐of‐the‐art saturation strategies. Furthermore, in vivo measurements were employed to demonstrate that OC saturation benefits extend to physiological conditions. The proposed pulses are integrated into the open‐source Pulseq‐CEST framework, providing an easily accessible alternative to conventional saturation pulses.

## Conflicts of Interest

The authors declare no conflicts of interest.

## Supporting information




**Data S1**: Supporting Information.

## Data Availability

The function integrating the OC saturation is provided in the Pulseq‐CEST library, including all mentioned OC pulses, example simulations and suggested usage: https://pulseq‐cest.github.io/.

## References

[1] D. Schache , A. Peddi , A. Nahardani , C. Faber , and V. Hoerr , “Corrections for Rabi Oscillations in Cardiac Chemical Exchange Saturation Transfer MRI Under the Influence of Very Short Preparation Pulses,” NMR in Biomedicine 37 (2024): e5081. 38113906 10.1002/nbm.5081

[2] J. R. Schüre , S. Weinmüller , L. Kamm , K. Herz , and M. Zaiss , “Sidebands in Cest Mr‐How to Recognize and Avoid Them,” Magnetic Resonance in Medicine 91 (2024): 2391–2402. 38317286 10.1002/mrm.30011

[3] P. Z. Sun , E. Wang , J. S. Cheung , X. Zhang , T. Benner , and A. G. Sorensen , “Simulation and Optimization of Pulsed Radio Frequency Irradiation Scheme for Chemical Exchange Saturation Transfer (CEST) MRI‐Demonstration of pH‐Weighted Pulsed‐Amide Proton CEST MRI in an Animal Model of Acute Cerebral Ischemia,” Magnetic Resonance in Medicine 66 (2011): 1042–1048. 21437977 10.1002/mrm.22894 PMC3135736

[4] R. J. Harris , T. F. Cloughesy , L. M. Liau , et al., “Simulation, Phantom Validation, and Clinical Evaluation of Fast Ph‐Weighted Molecular Imaging Using Amine Chemical Exchange Saturation Transfer Echo Planar Imaging (Cest‐Epi) in Glioma at 3 t,” NMR in Biomedicine 29 (2016): 1563–1576. 27717216 10.1002/nbm.3611

[5] Z. Mohanta , J. Stabinska , A. A. Gilad , P. B. Barker , and M. T. McMahon , “The Proton Resonance Enhancement for Cest Imaging and Shift Exchange (Precise) Family of Rf Pulse Shapes for Chemical Exchange Saturation Transfer MRI,” BioRxiv. 2024.06.19.599565 (2024). 10.1002/mrm.30410 PMC11893253 39831452

[6] C. Stilianu , C. Graf , M. Huemer , et al., “Enhanced and Robust Contrast in Cest MRI: Saturation Pulse Shape Design via Optimal Control,” Magnetic Resonance in Medicine 92 (2024): 1867–1880. 38818538 10.1002/mrm.30164

[7] V. Roeloffs , C. Meyer , P. Bachert , and M. Zaiss , “Towards Quantification of Pulsed Spinlock and Cest at Clinical Mr Scanners: An Analytical Interleaved Saturation–Relaxation (Isar) Approach,” NMR in Biomedicine 28 (2015): 40–53. 25328046 10.1002/nbm.3192

[8] W. T. Dixon , I. Hancu , S. J. Ratnakar , A. D. Sherry , R. E. Lenkinski , and D. C. Alsop , “A Multislice Gradient Echo Pulse Sequence for Cest Imaging,” Magnetic Resonance in Medicine 63 (2010): 253–256. 19918889 10.1002/mrm.22193 PMC2925508

[9] M. Huemer , C. Stilianu , and R. Stollberger , “Transient Model Based CEST Imaging ‐ tCEST,” Magnetic Resonance Materials in Physics 37 (2024): S31–S33.

[10] M. Huemer , C. Stilianu , and R. Stollberger , “Fully Transient 3d Model Based Cest,” in Proceedings of the CEST Conference 2024 (Friedrich‐Alexander University Erlangen‐Nürnberg, 2024).

[11] M. Huemer , C. Stilianu , and R. Stollberger , “Simultaneous Measurement of Cest and t1 Using Model‐Based Multi‐Pool‐Lorentzian Look‐Locker Reconstruction,” in Proceedings of the 2024 ISMRM & ISMRT Annual Meeting & Exhibition, Singapore (ISMRM Society, 2024), 4477.

[12] G. Liu , M. M. Ali , B. Yoo , M. A. Griswold , J. A. Tkach , and M. D. Pagel , “Paracest MRI With Improved Temporal Resolution,” Magnetic Resonance in Medicine 61 (2009): 399–408. 19165903 10.1002/mrm.21863 PMC4877027

[13] A. Liebert , M. Zaiss , R. Gumbrecht , et al., “Multiple Interleaved Mode Saturation (Mimosa) for b1+ Inhomogeneity Mitigation in Chemical Exchange Saturation Transfer,” Magnetic Resonance in Medicine 82 (2019): 693–705. 31002432 10.1002/mrm.27762

[14] D. Leitão , R. TomiTricot , P. Bridgen , et al., “Parallel Transmit Pulse Design for Saturation Homogeneity (Push) for Magnetization Transfer Imaging at 7t,” Magnetic Resonance in Medicine 88 (2022): 180–194. 35266204 10.1002/mrm.29199 PMC9315051

[15] Y. Völzke , S. Akbey , D. Löwen , et al., “Calibration‐Free Whole‐Brain Cest Imaging at 7t With Parallel Transmit Pulse Design for Saturation Homogeneity Utilizing Universal Pulses (Pushup),” Magnetic Resonance in Medicine 93 (2025): 630–642. 39301770 10.1002/mrm.30305 PMC11604840

[16] G. Rancan , T. Nguyen , and S. Glaser , “Gradient Ascent Pulse Engineering for Rapid Exchange Saturation Transfer,” Journal of Magnetic Resonance 252 (2015): 1–9. 25635353 10.1016/j.jmr.2014.12.016

[17] E. S. Yoshimaru , E. A. Randtke , M. D. Pagel , and J. CárdenasRodríguez , “Design and Optimization of Pulsed Chemical Exchange Saturation Transfer MRI Using a Multiobjective Genetic Algorithm,” Journal of Magnetic Resonance 263 (2016): 184–192. 26778301 10.1016/j.jmr.2015.11.006 PMC4871615

[18] T. Jin and S. G. Kim , “Advantages of Chemical Exchange‐Sensitive Spin‐Lock (CESL) Over Chemical Exchange Saturation Transfer (CEST) for Hydroxyl–and Amine–Water Proton Exchange Studies,” NMR in Biomedicine 27 (2014): 1313–1324. 25199631 10.1002/nbm.3191 PMC4201909

[19] K. Herz , C. Gandhi , M. Schuppert , A. Deshmane , K. Scheffler , and M. Zaiss , “CEST Imaging at 9.4 T Using Adjusted Adiabatic Spin‐Lock Pulses for On‐and Off‐Resonant t1ρ‐Dominated z‐Spectrum Acquisition,” Magnetic Resonance in Medicine 81 (2019): 275–290. 30194742 10.1002/mrm.27380

[20] K. Herz , T. Lindig , A. Deshmane , et al., “T1ρ‐Based Dynamic Glucose‐Enhanced (dgeρ) MRI at 3 t: Method Development and Early Clinical Experience in the Human Brain,” Magnetic Resonance in Medicine 82 (2019): 1832–1847. 31231853 10.1002/mrm.27857

[21] P. Schuenke , C. Koehler , A. Korzowski , et al., “Adiabatically Prepared Spin‐Lock Approach for t1ρ‐Based Dynamic Glucose Enhanced MRI at Ultrahigh Fields,” Magnetic Resonance in Medicine 78 (2017): 215–225. 27521026 10.1002/mrm.26370

[22] T. Jin , H. Mehrens , K. S. Hendrich , and S. G. Kim , “Mapping Brain Glucose Uptake With Chemical Exchange‐Sensitive Spin‐Lock Magnetic Resonance Imaging,” Journal of Cerebral Blood Flow & Metabolism 34 (2014): 1402–1410. 24865996 10.1038/jcbfm.2014.97 PMC4126103

[23] T. Jin , B. Iordanova , T. K. Hitchens , et al., “Chemical Exchange–Sensitive Spin‐Lock (Cesl) MRI of Glucose and Analogs in Brain Tumors,” Magnetic Resonance in Medicine 80 (2018): 488–495. 29569739 10.1002/mrm.27183 PMC5910214

[24] P. BoehmSturm , P. Schuenke , M. Foddis , et al., 2‐Deoxy‐D‐Glucose Chemical Exchange‐Sensitive Spin‐Lock MRI of Cerebral Glucose Metabolism After Stroke in the Rat. bioRxiv, 2025‐1 (2025). 10.1177/0271678X251355049 PMC12237932 40626496

[25] P. Schuenke , D. Paech , C. Koehler , et al., “Fast and Quantitative t1ρ‐Weighted Dynamic Glucose Enhanced MRI,” Scientific Reports 7 (2017): 42093. 28169369 10.1038/srep42093 PMC5294399

[26] P. S. Boyd , J. Breitling , F. Zimmermann , et al., “Dynamic Glucose‐Enhanced (Dge) MRI in the Human Brain at 7 t With Reduced Motion‐Induced Artifacts Based on Quantitative r1ρ Mapping,” Magnetic Resonance in Medicine 84 (2020): 182–191. 31788870 10.1002/mrm.28112

[27] K. Chow , P. Kellman , B. S. Spottiswoode , et al., “Saturation Pulse Design for Quantitative Myocardial t1 Mapping,” Journal of Cardiovascular Magnetic Resonance 17 (2015): 84. 26428468 10.1186/s12968-015-0187-0 PMC4589956

[28] M. Gram , M. Seethaler , D. Gensler , J. Oberberger , P. M. Jakob , and P. Nordbeck , “Balanced Spin‐Lock Preparation for b1‐Insensitive and b0‐Insensitive Quantification of the Rotating Frame Relaxation Time t1ρ ,” Magnetic Resonance in Medicine 85 (2021): 2771–2780. 33166009 10.1002/mrm.28585

[29] K. J. Layton , S. Kroboth , F. Jia , et al., “Pulseq: A Rapid and Hardware‐Independent Pulse Sequence Prototyping Framework,” Magnetic Resonance in Medicine 77 (2017): 1544–1552. 27271292 10.1002/mrm.26235

[30] K. Herz , S. Mueller , O. Perlman , et al., “Pulseq‐Cest: Towards Multi‐Site Multi‐Vendor Compatibility and Reproducibility of CEST Experiments Using an Open‐Source Sequence Standard,” Magnetic Resonance in Medicine 86 (2021): 1845–1858. 33961312 10.1002/mrm.28825 PMC9149651

[31] B. T. Polyak and A. B. Juditsky , “Acceleration of Stochastic Approximation by Averaging,” SIAM Journal on Control and Optimization 30 (1992): 838–855.

[32] D. Ruppert , “Efficient Estimations from a Slowly Convergent Robbins‐Monro Process, Technical report, Cornell University Operations Research and Industrial Engineering,” (1988).

[33] A. Rund , C. S. Aigner , K. Kunisch , and R. Stollberger , “Magnetic Resonance RF Pulse Design by Optimal Control With Physical Constraints,” IEEE Transactions on Medical Imaging 37 (2017): 461–472. 28981407 10.1109/TMI.2017.2758391

[34] J. P. Wansapura , S. K. Holland , R. S. Dunn , and W. S. BallJr , “Nmr Relaxation Times in the Human Brain at 3.0 Tesla,” Journal of Magnetic Resonance Imaging 9 (1999): 531–538. 10232510 10.1002/(sici)1522-2586(199904)9:4<531::aid-jmri4>3.0.co;2-l

[35] M. Zaiss , B. Schmitt , and P. Bachert , “Quantitative Separation of CEST Effect From Magnetization Transfer and Spillover Effects by Lorentzian‐Line‐Fit Analysis of z‐Spectra,” Journal of Magnetic Resonance 211 (2011): 149–155. 21641247 10.1016/j.jmr.2011.05.001

[36] P. Schuenke , J. Windschuh , V. Roeloffs , M. E. Ladd , P. Bachert , and M. Zaiss , “Simultaneous Mapping of Water Shift and b1 (Wasabi)‐application to Field‐Inhomogeneity Correction of Cest MRI Data,” Magnetic Resonance in Medicine 77 (2017): 571–580. 26857219 10.1002/mrm.26133

[37] C. Stilianu , A. Chubarov , S. Weinmüller , M. Huemer , M. Zaiss , and R. Stollberger , “Creatine and Phosphocreatine Cest Contrast of an Optimal Control Pulse Train Compared to an Optimized Gaussian Pulse Train for Cest Imaging in Calf Muscle,” Abstract Booklet European Society for Magnetic Resonance in Medicine and Biology (ESMRMB) 39 (2023): S86.

[38] J. Breitling , A. Deshmane , S. Goerke , et al., “Adaptive Denoising for Chemical Exchange Saturation Transfer Mr Imaging,” NMR in Biomedicine 32 (2019): e4133. 31361064 10.1002/nbm.4133

[39] C. Papageorgakis , M. Zucchelli , O. Dispasquale , et al., “Decorrelation Algorithm for Correcting b1 Artifacts in Aptw Imaging at 3 Tesla,” Proceedings of the 2024 ISMRM & ISMRT Annual Meeting & Exhibition, Singapore (2024): 3329.

[40] J. Zhou , M. Zaiss , L. Knutsson , et al., “Review and Consensus Recommendations on Clinical Apt‐Weighted Imaging Approaches at 3t: Application to Brain Tumors,” Magnetic Resonance in Medicine 88 (2022): 546–574. 35452155 10.1002/mrm.29241 PMC9321891

[41] A. Mennecke , K. M. Khakzar , A. German , et al., “7 Tricks for 7 t Cest: Improving the Reproducibility of Multipool Evaluation Provides Insights Into the Effects of Age and the Early Stages of Parkinson's Disease,” NMR in Biomedicine 36 (2023): e4717. 35194865 10.1002/nbm.4717

[42] F. Kogan , M. Haris , C. Debrosse , et al., “In Vivo Chemical Exchange Saturation Transfer Imaging of Creatine (Crcest) in Skeletal Muscle at 3t,” Journal of Magnetic Resonance Imaging 40 (2014): 596–602. 24925857 10.1002/jmri.24412 PMC4059780

[43] F. Kogan , M. Haris , A. Singh , et al., “Method for High‐Resolution Imaging of Creatine in Vivo Using Chemical Exchange Saturation Transfer,” Magnetic Resonance in Medicine 71 (2014): 164–172. 23412909 10.1002/mrm.24641 PMC3725192

[44] C. Stilianu , M. Huemer , and R. Stollberger , “Generalized Representation of Optimal Control CEST Saturation Pulses,” Magnetic Resonance Materials in Physics 37 (2024): S213–S215.

[45] B. Wu , G. Warnock , M. Zaiss , et al., “An Overview of Cest MRI for Non‐Mr Physicists,” EJNMMI Physics 3 (2016): 1–21. 27562024 10.1186/s40658-016-0155-2 PMC4999387

[46] Z. Zu , K. Li , V. A. Janve , M. D. Does , and D. F. Gochberg , “Optimizing Pulsed‐Chemical Exchange Saturation Transfer Imaging Sequences,” Magnetic Resonance in Medicine 66 (2011): 1100–1108. 21432903 10.1002/mrm.22884 PMC3151337

[47] P. C. VanZijl , W. W. Lam , J. Xu , L. Knutsson , and G. J. Stanisz , “Magnetization Transfer Contrast and Chemical Exchange Saturation Transfer MRI. Features and Analysis of the Field‐Dependent Saturation Spectrum,” NeuroImage 168 (2018): 222–241. 28435103 10.1016/j.neuroimage.2017.04.045 PMC5650949

